# Intraocular Temperature Distribution in Eyes Undergoing Different Types of Surgical Procedures during Vitreous Surgery

**DOI:** 10.3390/jcm11072053

**Published:** 2022-04-06

**Authors:** Kei Shinoda, Soiti C. Matsumoto, Kazuma Yagura, Gaku Terauchi, Takuhei Shoji, Yuji Yoshikawa, Yuro Igawa, Atsushi Mizota, Yozo Miyake

**Affiliations:** 1 Department of Ophthalmology, Teikyo University School of Medicine, Itabashi-ku, Tokyo 173-8605, Japan; soiti@icloud.com (S.C.M.); yagpiero@yahoo.co.jp (K.Y.); jakkaruniomakase22@yahoo.co.jp (G.T.); mizota-a@med.teikyo-u.ac.jp (A.M.); 2 Department of Ophthalmology, Saitama Medical University Faculty of Medicine, Iruma-gun, Saitama 350-0495, Japan; shoojii@gmail.com (T.S.); yuji.yoshi.md@gmail.com (Y.Y.); 22bonobono22@gmail.com (Y.I.); 3 Matsumoto Eye Clinic, Awa, Tokushima 771-1705, Japan; 4 Kobe City Eye Hospital, Kobe, Hyogo 650-0047, Japan; ymiyake@aichi-med-u.ac.jp

**Keywords:** vitreous surgery, intraocular temperature, temperature gradient

## Abstract

Vitreous temperature has been reported to vary during intraocular surgery. We measured the temperature at three intraocular sites, just posterior to the crystalline lens (BL), mid-vitreous (MV), and just anterior to the optic disc (OD), and investigated temperature changes before and after different types of surgical procedures in 78 eyes. The mean temperature at the beginning was 30.1 ± 1.70 °C in the anterior chamber, 32.4 ± 1.41 °C at the BL, 33.8 ± 0.95 °C at the MV, and 34.7 ± 0.95 °C at the OD. It was lowest at the BL, and highest at the OD. The mean temperature after cataract surgery was slightly lower especially at an anterior location. Thus, the temperature gradient became slightly flatter. The mean temperature after core vitrectomy was even lower at all sites and a gradient of the temperature was not present. The mean temperature after membrane peeling was significantly higher than that after core vitrectomy, and there was no gradient. The mean temperature after fluid/air exchange was lower at the BL and higher at the MV and at the OD. Thus, a gradient of higher temperatures at the OD appeared. The intraocular temperature distribution is different depending on the surgical procedure which can then change the temperature gradient. The temperature changes at the different intraocular sites and the gradients should be further investigated because they may affect the physiology of the retina and the recovery process.

## 1. Introduction

Vitreous surgery has been performed since the 1970s, and its effectiveness has greatly increased with the introduction of new techniques and instruments [1,2]. During vitreous surgery, the intraocular environment is altered by the procedures, and the intraocular tissues such as the retina can be exposed to non-physiological temperatures. Electroretinographic monitoring of the retinal function during vitrectomy [3,4,5,6,7] showed that the whole and local retinal function varied depending on the surgical procedures, and the variations were partly related to the retinal temperatures [4]. Furthermore, it was recently reported that changes in the temperature of the ocular surface are correlated with changes in retinal blood flow [8]. In addition, the temperature of the ocular surface differs depending on the retinal disease [9]. Therefore, it is expected that the temperature of the environment surrounding the retina will affect the blood flow and function of the physiological and pathological retina.

Alio and Pardon were the first to measure the temperature of both orbito-ocular areas in a normal subject in 1982 [10]. Recently, studies measuring the intraocular temperature in a specific location of the vitreous cavity during vitreous surgery have reported changes in the temperature during various procedures [11,12,13]. Landers et al. [11] measured different regions of the retinal surface as well as the mid-vitreous temperatures before, during, and after vitrectomy in six eyes that underwent 23G pars plana vitrectomy (PPV). They found that the vitreous cavity and retina were cooled during vitrectomy to much lower temperatures than those used in therapeutic hypothermia [14,15,16] and increased at the end of vitrectomy. In 2013, Romano et al. [17] measured the intraocular temperature continuously in 14 eyes during 25G PPV. They also measured the temperature of the surface of the nasal retina. However, to the best of our knowledge, there has not been a study of the temperature at different sites of the vitreous before and after various types of intraocular surgery.

Thus, the purpose of this study was to determine the temperature at three intraocular sites before and after several procedures during vitrectomy. The temperature of the anterior chamber (AC) was also measured in some the eyes. We used a thermoprobe to measure the temperature in the AC, just behind the crystalline lens or intraocular lens (IOL) (BL), the mid-vitreous (MV), and just anterior to the optic disc (OD). We analyzed the relationship between the type of intraocular surgical procedure to the temperature at the four sites.

## 2. Patients and Methods

### 2.1. Patients

The participants were 78 patients who underwent vitrectomy for the first time as part of their treatment between November 2013 and July 2016 at the Teikyo University Hospital in Tokyo, Japan. The procedures used were approved by the Institutional Review Board of the Teikyo University (ID:10-033-2), and a written informed consent was obtained from all the participants.

The clinical characteristics of the 78 patients are shown in Table 1. Fifty-three of the patients were men, and 25 were women, and their average age was 63.4 ± 12.8 (±SD) years with a range from 34 to 87 years. Eighteen eyes were pseudophakic, 12 eyes had a clear crystalline lens, and 48 eyes had cataracts for which combined cataract surgery and vitrectomy were performed. A peeling of an epiretinal membrane (ERM) or the internal limiting membrane was performed in 36 eyes for the treatment of an ERM, macular hole (MH), or macular edema, or to prevent ERM formation during surgery for rhegmatogenous retinal detachment (RRD) or proliferative diabetic retinopathy (PDR). Fluid/air exchange was performed in 29; all eyes with a MH or RRD and for some eyes with PDR or ERM that were performed at the surgeon’s discretion. The indications for the vitrectomy are listed in Table 1.

### 2.2. Temperature Measurements

Temperature measurements were made with a 26-gauge flexible wire thermoprobe with the temperature sensor located at the tip of the probe. (Needle Microprobe MT-26/6, Physitemp Instruments LLC, Clifton, NJ, USA). This probe was used in previous studies to measure the intraocular temperature [18,19]. It is a Fast-response, 26-gauge needle probe, (needle length, 4 cm) for instant recordings in tissues, semisolids, and liquids. The needle tip is sealed to ensure that only stainless-steel surfaces contact the specimens. The probe can measure a maximum temperature of 200 °C and a minimum temperature of −50 °C. The time constant of the probe was 0.1 s (https://physitemp.com/needmicroprob_p157, accessed on 1 February 2022)). The thermoprobe is accurate to within ±0.1 °C and equilibrates with the surrounding fluid within 2 to 3 s. The thermoprobe was sterilized in ethylene oxide gas before use.

The microprobe was connected to an analogue digital converter (PL3508 PowerLab 8/35, AD Instruments, Bella Vista, New South Wales, Australia), and data analyses were performed by the LabChart Pro software (AD instruments, Bella Vista, New South Wales, Australia). The tip of the probe was introduced through a trocar and placed at the three intravitreal sites, the BL, MV, and OD. To measure the temperature in the AC, the tip of the thermoprobe was inserted into the AC through a preplaced corneoscleral side port. Immediately after the temperature in the AC was measured, the thermoprobe was withdrawn and inserted into the vitreous cavity through a preplaced trocar.

The temperature measurements were made just after the trocars were placed and with the inflow and outflow of the infusion channel turned off, just after the core vitrectomy with the inflow and outflow channel on, just after the membrane peeling with the inflow and outflow channel off, and during fluid/air exchange with the inflow and outflow channel on. In eyes that underwent phacovitrectomy, the temperature measurements were made just after the cataract surgery with the inflow and outflow channel off.

The room temperature was set at 25 °C, and the temperature of the patient’s skin was 36.3 ± 0.4 °C. The surgeon and surgical assistant localized the tip of the thermoprobe under observation through the operating microscope with binocular indirect OphthalmoMicroscope (OCULUS Optikgeräte GmbH, Wetzlar, Germany) and a wide-angle observation system for the vitreous surgery. Another assistant monitored the temperature changes in real time on a personal computer, and he notified the surgeon when the temperature had stabilized. This allowed for rapid and accurate measurements and minimized the local fluctuations. The total time required to record these measurements was approximately 1 min.

All surgeries were performed with the CONSTELLATION^®^ Vision System (Alcon, Fort Worth, TX, USA) with 25-gauge instruments, and the surgeries were performed under sub-Tenon anesthesia. After prepping and draping in a sterile fashion, valved trocars were placed at the conventional sites for pars plana standard 25-gauge vitrectomy.

### 2.3. Statistical Analyses

Comparisons of the temperature at the four different sites was carried out using analysis of variance followed by a post hoc test (Tukey–Kramer test). In addition, a linear mixed model was used to analyze the ocular data because repeatable measurements from 1 eye and measurements from both eyes of the same individual are likely to be highly correlated. The linear mixed method was also used to adjust for the within-individual repeated measurements correlations. This type of analysis resulted in the most efficient use of the data while considering the high level of correlations among the repeated measurements in the same eye. We analyzed the relationship between the temperature and variables such as measurement timing and measurement intraocular site using a linear mixed model, whereby each eye was treated as a random effect. The JMP version 10.1 software (SAS Institute Inc., Cary, NC, USA) and Stata software version 15 (StataCorp LP, College Station, TX, USA) were used for the statistical analyses. A *p* <0.05 was taken to be statistically significant.

## 3. Results

The temperature measurements were made just before and just after membrane peeling in 36 eyes and after fluid/air exchange in 14 eyes. The temperature measurements were not made in some eyes undergoing fluid/air exchange because the fundus visibility was poor or because perfluorocarbon liquid was used to tamponade the retina. In the 48 eyes that had undergone phacovitrectomy, the temperature measurements were made just after the cataract surgery with the inflow and outflow channel turned off.

**Figure 1:**
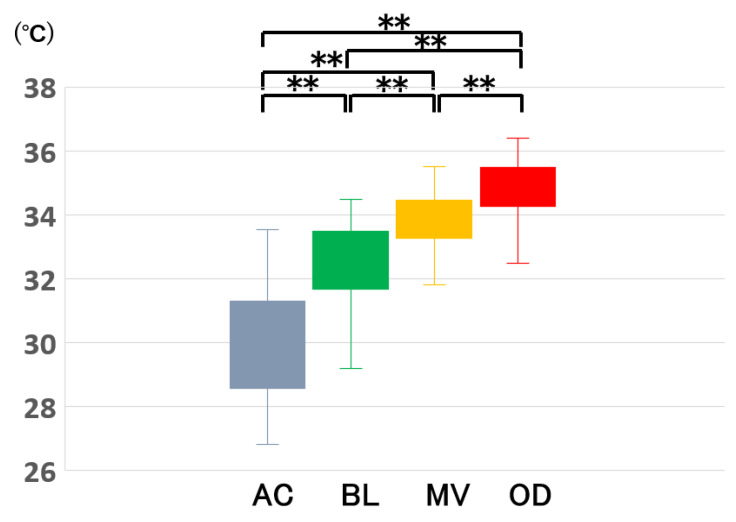
Intraocular temperature distribution at the beginning of surgery. There was a significant difference in the temperature between any two of the four sites. The temperature was lowest in the anterior part of the eye, and it gradually increased at sites closer to the posterior retina. Thus, there was a gradient with higher temperatures toward the posterior of the eye. The upper end of the whisker is the maximum value, and the lower end is the minimum value. The box indicates the interquartile range; the horizontal line in the box indicates the median value, and the × indicates the mean. AC, anterior chamber; BL, just posterior to the crystalline lens or intraocular lens; MV, mid-vitreous; OD, just anterior to the optic disc. **: *p* < 0.01.

The means ± SDs and the [medians (interquartile range)] of the intraocular temperature was 30.1 ± 1.70 °C [30.6 (28.6, 31.3 °C)] in the AC, 32.4 ± 1.41 °C [32.6 (31.7, 33.5 °C)] at the BL, 33.8 ± 0.95 °C [33.8 (33.3, 34.5 °C)] at the MV, and 34.7 ± 0.95 °C [34.7 (34.3, 35.5 °C)] at the OD (Figure 1). The difference in the temperatures between any two of the four sites was significant. Thus, the temperature was lowest in the anterior part of the eye, and it gradually increased to that at the OD. Interestingly, the temperature at the OD was lower than the body temperature of 36.3 ± 0.4 °C.

The mean temperature immediately after cataract surgery was slightly lower than the presurgical temperature especially in the anterior location at 29.9 ± 1.31 °C [29.9 (28.9, 30.7 °C)] at the BL, 32.1 ± 1.36 °C [32.3 (31.2, 33.4 °C)] at the MV, and 33.8 ± 1.74 °C [34.1 (33.5, 34.9 °C)] at the OD. Thus, the temperature was highest close to the OD, and the gradient was slightly flatter.

The mean temperature soon after core vitrectomy was lower at 29.5 ± 1.94 °C [29.3 (28.3, 30.9 °C)] at the BL, 29.6 ± 2.01 °C [29.5 (28.3, 30.9 °C)] at the MV, and 30.0 ± 1.92 °C [30.1 (28.7, 31.7 °C)] at the OD. Under these conditions, the gradient was flat. The mean temperature after membrane peeling was significantly higher at 32.1 ± 1.88 °C [32.2 (30.3, 33.7 °C)] at the BL, 32.6 ± 1.77 °C [32.7 (31.4, 34.1 °C)] at the MV, and 32.6 ± 1.87 °C [32.8 (31.1, 34.2 °C)] at the OD. The gradient was flat. The mean temperature after fluid/air exchange was 30.2 ± 2.38 °C [31.1(27.7, 32.0 °C)] at the BL, 33.0 ± 1.36 °C [33.1 (31.7, 34.1 °C)] at the MV, and 34.3 ± 1.14 °C [34.6 (33.7, 35.3 °C)] at the OD. The temperature was lower at the BL and higher at the OD with higher temperatures toward the OD.

**Figure 2:**
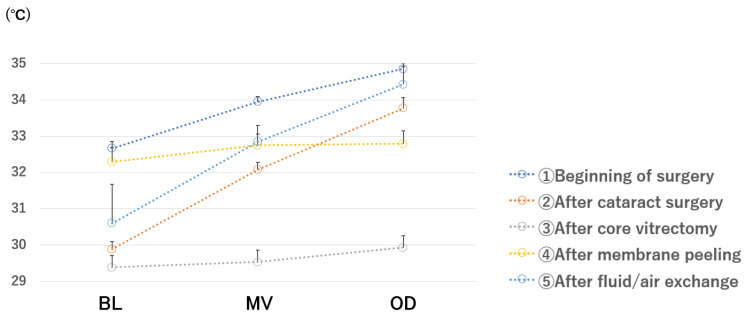
Local temperatures at several intraocular sites. 1 and 2: A temperature gradient was present prior to the surgery, and it became flatter after cataract surgery. 3: After core vitrectomy, the gradient is not present, and the temperature gradient is flattened. 4: After membrane peeling, a temperature gradient is not present. 5: After fluid/air exchange, the temperature is higher at the posterior site and lower at the anterior site, and the temperature gradient is present. Eighteen eyes that did not undergo cataract surgery were excluded. BL, just behind the intraocular lens; MV, mid-vitreous; OD, just anterior to the optic disc.

The local temperatures at several intraocular sites are shown in Figure 2. The temperature gradient was present at the beginning, and it became flatter after cataract surgery. After core vitrectomy, a gradient was not present. A steeper gradient was detected after membrane peeling, and a gradient was present after fluid/air exchange.

**Figure 3:**
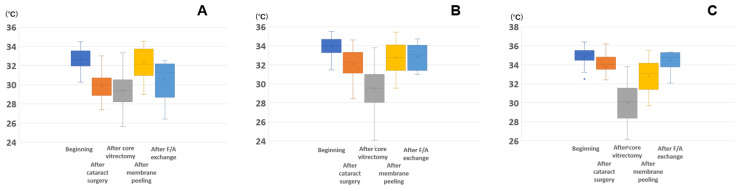
Intraocular temperature changes at a specific site during vitrectomy: (**A**) just posterior to the intraocular lens (BL), (**B**) mid-vitreous (MV), and (**C**) just anterior to the optic disc (OD). (**A**) The temperature at the BL is lower after cataract surgery and was stable after core vitrectomy and higher after membrane peeling. (**B**) The temperature at the MV is lower after cataract surgery and further lower after core vitrectomy and higher after membrane peeling and was stable after F/A exchange. (**C**) The temperature at the OD is lower after cataract surgery and is stable after core vitrectomy and higher after membrane peeling and tended to further increase after F/A exchange. Number of eyes: beginning (*n* = 48), after cataract surgery (*n* = 46), after core vitrectomy (*n* = 41), after membrane peeling (*n* = 36), after F/A exchange (*n* = 10). The upper end of the whisker is the maximum value, whereas the lower end is the minimum value; the *box* indicates the interquartile range; the *line in the box* indicates the median value, the × indicates the mean, and the closed circle indicates the outlier. F/A, fluid/air exchange.

The intraocular temperatures at the three sites before and after the different surgical procedures are plotted in Figure 3, showing that the temperature at the three sites changed differently. BL was lower after cataract surgery and was not changed after core vitrectomy and was higher after membrane peeling (Figure 3A). The temperature at the MV was lower after cataract surgery and even lower after core vitrectomy, and it was higher after membrane peeling and was stable after fluid/air exchange (Figure 3B). The temperature at the OD was lower after cataract surgery and was unchanged after core vitrectomy and was higher after membrane peeling and tended to be higher after F/A exchange (Figure 3C). Table 2 shows the factors associated with the temperature using a linear mixed model. Both the timing of the measurements and intraocular sites were significantly and independently associated with the temperature. The temperature at the beginning of surgery was significantly higher than other timing of measurement (all *p* < 0.001). A trend towards higher temperatures in deeper intraocular locations was observed (*p* < 0.001).

## 4. Discussion

The effects of temperature on vitrectomy have been extensively investigated in animal studies, and lower temperatures have been shown to reduce the breakdown of the blood–water barrier and reduce intraocular bleeding and postoperative inflammation [20,21]. In addition, electroretinography during vitrectomy suggested that changes in the temperature of the irrigating fluid may temporarily alter the retinal physiology [4].

In vitreous surgery, the temperature of the retina changes depending on various factors such as the specific heat and temperature of the substance that fills the vitreous cavity and the content of the surgical procedure and its duration. For example, prolonged photocoagulation raises the temperature of the retina [12], and when replaced with air, dryness and temperature changes can have some effect on the retina [22]. In addition, substances and temperature in the vitreous cavity affect the diffusion and convection of metabolites, electrolytes, and cytokines.

In patients with intracerebral hemorrhage, systemic cooling target temperatures ranged from 30 to 33 °C, while local cooling target temperatures ranged from 11 to 33 °C [23]. Hypothermia was reported to be an effective treatment for traumatic retinal injury [24]. However, there are no clear recommendations regarding the safety of specific temperature conditions in the intraocular environment during vitreoretinal surgery.

In this study, we measured the intraocular temperature at different sites before vitreous surgery and found that the temperature distribution changed immediately after several procedures during the vitreous surgery. There was a temperature gradient with the temperature lowest at the BL and highest at the OD. In addition, the steepness of the gradient changed or even reversed depending on the surgical procedures.

The metabolic activity and blood flow in the posterior segment of the eye is one of the major factors affecting the intraocular temperature. The temperatures of the retina, choroid, sclera, and bulbar conjunctiva of cats and monkeys have been reported to be lower following a decrease of choroidal blood flow [25]. Patients with reduced ocular circulation, such as carotid artery stenosis, have been reported to have reduced external ocular surface temperatures [26,27].

Our results showed that at the beginning of the surgery, the highest temperature was at the OD although it was still lower than the body temperature. This is in accordance with a previous study that measured mid-vitreous temperature during phacoemulsification and found that the temperature was lower than the body temperature, and it decreased significantly from the baseline after each surgical step and surgical time [13,28]. Our results showed that the temperature decreased even at the OD, although it decreased more at the anterior sites; hence a different temperature gradient was present after cataract surgery.

A temperature gradient was not present after core vitrectomy. This was most likely due to the uniform spread of the irrigation fluid throughout the vitreous cavity.

The intraocular temperature after membrane peeling was higher than that after core vitrectomy at each site, and the gradient was not present. Iguchi et al. also reported that the mid-vitreous temperature increased during membrane peeling, and its change did not depend on the duration of the surgery [12].

Interestingly, after fluid/air exchange, the temperature at the BL was lower, and that at the OD was higher than that before the exchange. This resulted in the presence of the temperature gradient. It is believed that the warming effect of the retinochoroidal circulation in the posterior fundus and the cooling effect of the room air temperature in the anterior part of the eyes played roles after this surgical procedure. This temperature gradient with air perfusion was different from that at the start of surgery. This is probably due to the difference in the specific heat property of the substance that fills the eye. We have measured the intraocular temperature in surgically naïve eyes and eyes that had a prior vitrectomy and found that the temperature gradient was different depending on the intravitreal material, e.g., formed or liquid vitreous or silicone oil [18].

The temperature changed during the surgery because of the influence of each surgical procedure (Figure 3). We suggest that the distance between the retinochoroid and the external ocular surface as well as the cooling effect of the irrigation fluid influenced the temperature changes.

We have recorded full-field electroretinograms (ERGs) and focal macular ERGs just after core vitrectomy and observed a reduction in the amplitude and prolongation of the implicit time in several ERG components that originated from different retinal layers [5,6]. Landers et al. reported [11] that the retinal surface temperature was 34.8–35.2 °C before, 28.4–29.5 °C during, and 32.3–32.7 °C after vitrectomy. These temperatures are within the range of the temperatures at the OD in our cohort. However, the temperature of the retina, especially the temperature in the different retinal layers, is not known. We measured the subretinal temperature by implanting a polyimide foil strip carrying an optical sensor as well as a thermal sensor into the subretinal space of the eyes of rabbits [29]. Our results showed an exponential relationship between the power of the infrared (IR) irradiation and the temperature increases with increasing IR irradiation power up to 12.7 mW/mm^2^ in the living animal. The maximum temperature increases plateaued at 4.5 °C. In addition, the temperature increased linearly at 1.15 °C/1 mW/mm^2^ of the IR irradiation density in postmortem eyes, i.e., eyes without ocular blood flow over 5.5 °C. The retina can be cooled immediately by the choroidal blood flow.

The ERG changes may also be related to other unidentified factors rather than just the temperature. Vitrectomy causes changes in electrolytes and osmolality as well as indirect mechanical changes of the retina which may contribute to the ERG changes [30,31,32]. Rinkoff et al. suggested that vitreoretinal surgeons should avoid warming irrigation solutions to levels above room temperature to minimize light damage to the retina [33].

Our study has several limitations. The number of eyes where the temperature measurements could be made after membrane peeling and during F/A exchange was relatively small. In addition, the pathology requiring the vitrectomy was not the same in all cases. In eyes with retinal circulatory disorders, such as diabetic retinopathy, the choroidal circulation may be altered which may explain the differences in the intraocular temperatures. Further investigations are needed to confirm the results with a larger number of eyes with the same similar retinal diseases. All the measurements were carried out when patients were in the supine position in the operating room where the temperature was set at 25 °C. Therefore, the interpretation of the results should be carefully carried out. The distribution of the temperatures may differ depending on the patient’s position, environmental temperature, and patients’ activity.

## 5. Conclusions

In conclusion, we measured the intraocular temperature at different sites during vitreous surgery. We found an intraocular temperature gradient with higher temperatures closer to the retina. The intraocular temperature distribution changes depended on the surgical procedures used during vitrectomy, and these procedures can steepen or flatten the temperature gradient. These findings may bring new insights into the physiological, pharmacological, and clinical consequences of vitrectomy. The temperature changes at the different intraocular sites and the gradient should be further investigated because they may affect the physiology of the retina and the recovery process.

## 6. Patents

No patents are included in this study.

## Figures and Tables

**Table 1:** Clinical characteristics of the patients.

Male/Female	53/25
age (years) (mean ± SD)	63.4 ± 12.8
body temperature (°C) (mean ± SD)	36.3 ± 0.4
lens status phakia/IOL	60/18
surgical indication	
PDR	22
ERM	20
RRD	15
MH	9
IOL dislocation	4
Terson	3
BRVO	3
DME	1
MacTel	1

**Table 2:** Association between the intraocular temperature and variables using multivariable analysis.

			Coef. (95% CI)	*p* Value
timing of the measurement (surgical procedure)	1	beginning	Ref.	
	2	after cataract surgery	−2.02 (−2.31, −1.73)	<0.001
	3	after core vitrectomy	−3.92 (−4.21, −3.63)	<0.001
	4	after membrane peeling	−1.44 (−1.8, −1.09)	<0.001
	5	after fluid/air exchange	−1.16 (−1.7, −0.63)	<0.001
intraocular site			1.38 (1.27, 1.49)	<0.001

## Data Availability

The data are not publicly available due to personal information protection.
